# Editorial: Unravelling molecular mechanisms of citrus resistance to huanglongbing: from effectors to engineered immunity, biomarkers, and deployment strategies

**DOI:** 10.3389/fpls.2026.1828457

**Published:** 2026-04-07

**Authors:** Raphael Morillon, Eduardo Augusto Girardi, Barbara Hufnagel

**Affiliations:** 1 Unité Mixte de Recherche Amélioration Génétique et Adaptation des Plantes méditerranéennes et tropicales (UMR AGAP) Institut, Univ. Montpellier, Centre de coopération Internationale en Recherche Agronomique pour le Développement (CIRAD), Institut National de Recherche pour l’Agriculture, l’Alimentation et l’Environnement (INRAE), Institut Agro, Montpellier, France; 2 Empresa Brasileira de Pesquisa Agropecuária (Embrapa), Embrapa Mandioca e Fruticultura, Cruz das Almas, Bahia, Brazil; 3 Fundo de Defesa da Citricultura (Fundecitrus), Araraquara, São Paulo, Brazil

**Keywords:** *Candidatus* Liberibacter asiaticus (CLas, CLaf, CLam), HLB (Huanglongbing), transcriptomic and metabolic dynamics, psyllids (*Diaphorina citri* and *Trioza erythreae*), salicylic acid

## Introduction

HLB (huanglongbing), largely associated with *Candidatus* Liberibacter asiaticus bacteria (CLas, CLaf, CLam) and transmitted by psyllids (*Diaphorina citri* and *Trioza erythreae*) remains one of the most damaging diseases of citrus worldwide. The eight studies assembled here collectively shift the focus from late, symptomatic snapshots towards early events, mechanistic causality, and actionable solutions, spanning vector- and pathogen-derived effectors, host transcriptomic and metabolic dynamics, salicylic acid-centred immune engineering, allele-specific regulatory architectures, and graft-based containment strategies. Together, they refine a key message: HLB outcomes are shaped not only by “defence activation”, but by how citrus controls phloem function, redox balance, hormone crosstalk, and metabolic suitability for CLas.

## Effectors as levers of disease and opportunities for control

**Figure 1:**
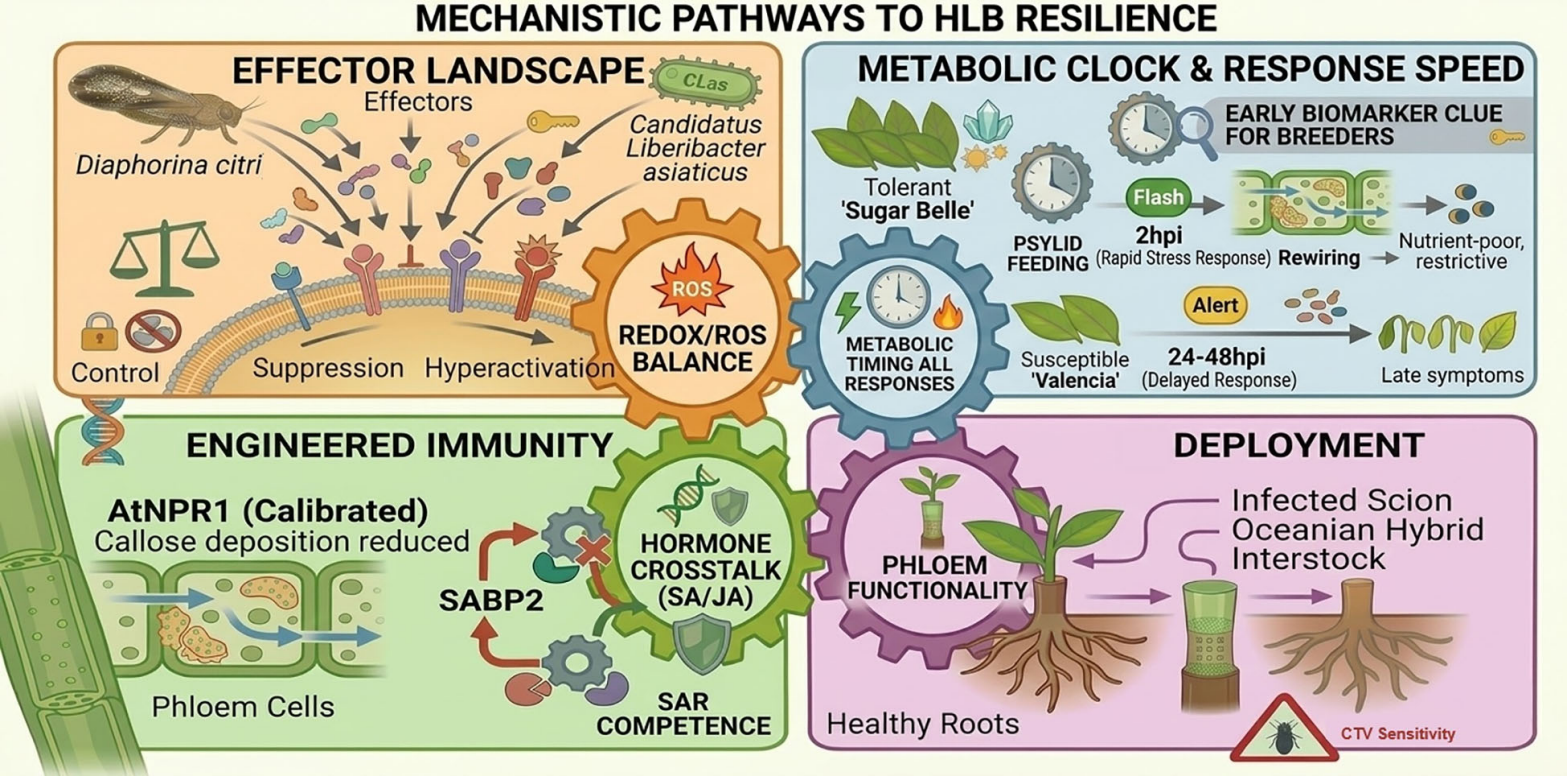
Mechanistic pathways associated with HLB resilience in citrus. Top left: Interaction between the vector (*Diaphorina citri*), *Candidatus* Liberibacter asiaticus (CLas) effectors, and citrus phloem cells, illustrating effector delivery and modulation of host redox responses. Bottom left: Molecular mechanisms contributing to systemic acquired resistance (SAR). Expression of AtNPR1 and activity of SABP2 influence defense signaling, hormonal crosstalk, and phloem-associated responses. Top right: Temporal dynamics of host metabolic responses following infection. The tolerant cultivar ‘Sugar Belle’ shows rapid metabolic activation and accumulation of defensive metabolites (e.g., flavonoids), whereas the susceptible cultivar ‘Valencia’ displays delayed responses. Bottom right: Grafting-based deployment strategies. Interstocks derived from Oceanian citrus relatives can restrict bacterial movement toward the root system, although some combinations may remain sensitive to Citrus tristeza virus (CTV).

A central advance is the functional identification of candidate effectors from both the vector and the bacterium (Dangol et al.). By combining psyllid head RNA-seq with proteomics of artificial diet and phloem exudates across multiple hosts, the study reveals a complex “effector landscape” present at the feeding interface (**Figure 1**, top-left). Importantly, transient expression assays showed that these candidates differentially modulate pathogen associated molecular-PTI outputs, particularly the flg22-induced ROS burst: ferritin and CLIBASIA_04560 suppressed ROS, while several psyllid proteins and CLIBASIA_05320 enhanced ROS. This bidirectional modulation is not a contradiction; rather, it highlights that either ROS suppression or ROS hyperactivation can be beneficial to the pathosystem, depending on timing, localisation, and downstream costs to the host.

The accompanying mini-review “Swords and shields” (Hu et al.) synthesises this concept by framing CLas Sec-dependent effectors and non-classically secreted proteins as multi-target virulence tools affecting SAR, ROS, vesicle trafficking, callose, autophagy, chlorosis, and flowering. A key implication emerging from both pieces is strategic: resistance breeding and engineering may be most effective when directed at conserved host “hubs” (susceptibility nodes) and effector targets, rather than chasing single symptom-linked genes.

## Early infection is not always “classical immunity”

A striking, and potentially paradigm-shifting, result comes from the transcriptomic analysis of early stages of CLas inoculation via *D. citri* feeding on young shoots (Alves et al.). Comparing susceptible *Citrus × sinensis* with resistant relatives (*Murraya paniculata*, transient host; *Bergera koenigii*, immune), the authors show that, despite successful bacterial delivery, few DEGs distinguish CLas-positive from CLas-negative exposures at matched time points. Moreover, canonical PTI/ETI signatures are not strongly enriched in the resistant species. Instead, the resistant hosts show subtle but consistent evidence for metabolic rewiring (e.g., early downregulation of photosynthesis-related processes and sulphur amino-acid pathways in *Bergera*), suggesting that host suitability (nutrient and metabolic “niche”) and inducible physiological reprogramming may restrict CLas multiplication.

This has two important consequences for the field: i) Early resistance may not look like “strong defence gene induction”, especially in phloem-limited pathosystems, ii) Experimental designs must carefully separate developmental transcriptome dynamics (dominant in young flushes) from pathogen-driven changes, otherwise false mechanistic conclusions become likely.

## Engineering SAR: NPR1 and SABP2 as complementary routes

Two studies converge on salicylic-acid-centred immune optimisation as an actionable route to tolerance.

NPR1-mediated early tolerance: in the NPR1-focused work (Sarkar et al.), AtNPR1 overexpression establishes a primed basal state (downregulation of cytoskeleton/cell-wall/receptor signalling pre-infection), followed by an earlier and stronger transcriptional response after CLas exposure (6–24 hours post infection, hpi). Notably, AtNPR1 plants suppress callose synthases and induce β-1, 3-glucanases by 24 hpi, implying a controlled balance between phloem sealing and phloem functionality. (**Figure 1**, bottom-left). The study also highlights ROS moderation and stabilised hormone crosstalk ((salicylic acid (SA), jasmonoyl-L-isoleucine (JA-Ile), gibberellin A8 (GA8), and abscisic acid (ABA)), reinforcing that tolerance may depend on preventing collateral damage as much as on activating defence.

SABP2 and breaking SA feedback inhibition: the SABP2 study (Dong et al.) adds a powerful mechanistic refinement, because a single substitution (V18A) removes SA feedback inhibition of SABP2 activity, enhancing MeSA→SA conversion and SAR competence. In transgenic sweet orange, only CsSABP2-1V18A reduced CLas titres/symptoms, while all SABP2 variants reduced canker lesions, indicating that tuning SA/MeSA homeostasis can confer multi-disease benefits (**Figure 1**, bottom-left). Together with NPR1-based findings, this supports a practical editorial conclusion: successful engineering is likely to require “calibrated immunity” (priming + controlled redox/hormone balance), not maximal defence activation.

## Metabolomics reveals speed and baseline capacity as hallmarks of tolerance

The temporal metabolomics study (Li et al.) demonstrates that the earliest metabolic responses to CLas can occur within hours, with the tolerant genotype (‘LB8-9’ Sugar Belle) responding as early as 2 hpi, compared with 24–48 hpi in susceptible ‘Valencia’ (**Figure 1**, top-right). Beyond timing, a key discriminator is *baseline capacity*: tolerant leaves maintain higher flavonoid pools across 48 h, while oxylipin-related pathways are enriched early. This suggests a two-component tolerance model: i) high constitutive protective metabolites (especially flavonoids), plus ii) rapid inducible activation of stress-related pathways. The practical value is immediate: these metabolites offer candidates for early-stage biomarkers, supporting non-destructive phenotyping pipelines and faster selection in breeding programs.

## Regulatory architecture: why hybrids tolerate and how?

The genetic architecture study in the tolerant *Citrus* × *Poncirus* hybrid US-897 (Diaz et al.) provides a framework for interpreting tolerance as an emergent property of regulatory inheritance. Most DEGs show expression-level dominance (~80%), allele-specific expression is widespread, and interspecific divergence is strongly shaped by cis-regulation. Interestingly, early infection-responsive genes show a reduced cis signature, implying a dynamic cis/trans interplay during stress. The identification of additive expression with cis-biased allelic contributions for SAR negative regulators (NPR3/NPR4) strengthens the idea that fine-scale regulatory tuning, rather than single major genes, may underpin durable tolerance in interspecific genotype.

## Deployment strategy: can resistant interstocks protect roots?

Finally, the interstock study (Darolt et al.) tackles a pressing translational question: can HLB-resistant Oceanian genotypes block CLas movement from an infected scion to susceptible roots? Under greenhouse conditions, scions were colonised regardless of interstock genotype, but root invasion was partially blocked in a substantial fraction of trees (up to 86% for specific Oceanian hybrid interstocks, **Figure 1**, bottom-right). Anatomical observations (smaller sieve pores; less occlusion) support a physical/functional contribution to reduced movement. However, the work also highlights real-world constraints, notably CTV sensitivity and stem pitting affecting horticultural performance. The message is nuanced but valuable: interstocks may not be a stand-alone cure, yet they could become part of integrated management, provided that field validation, optimisation (interstock length/top-working), and CTV-tolerant germplasm are addressed.

## Outlook: towards integrated, early-stage, mechanism-driven HLB solutions

Across these eight studies, three cross-cutting priorities emerge: i) Early, spatially resolved biology (phloem cell types; fast time scales) to avoid conflating development with infection, ii) Calibrated immunity (SAR priming, callose turnover control, moderated ROS/hormone balance) as a more reliable route to tolerance than indiscriminate defence induction, iii) Actionable translation via biomarkers, regulatory maps in hybrids, and graft-based strategies, each constrained by agronomic realities (CTV, performance, field durability). HLB research is thus moving from describing damage to engineering resilience, using mechanistic levers that are increasingly testable, comparable across systems, and deployable in breeding and biotechnology pipelines. Ultimately, although the specific mechanisms that explain plant responses to CLas and disease functioning remain unknown, the path to achieving this understanding is on the horizon.

